# Voxel-wise deviations from healthy aging for the detection of region-specific atrophy

**DOI:** 10.1016/j.nicl.2018.09.013

**Published:** 2018-09-19

**Authors:** Stefan Klöppel, Shan Yang, Elias Kellner, Marco Reisert, Bernhard Heimbach, Horst Urbach, Jennifer Linn, Stefan Weidauer, Tamara Andres, Maximilian Bröse, Jacob Lahr, Niklas Lützen, Philipp T. Meyer, Jessica Peter, Ahmed Abdulkadir, Sabine Hellwig, Karl Egger, for the Alzheimer's Disease Neuroimaging Initiative

**Affiliations:** a University Hospital of Old Age Psychiatry, University of Bern, Bern, Switzerland; b Freiburg Brain Imaging Center, Medical Center - University of Freiburg, Faculty of Medicine, University of Freiburg, Germany; c Center of Geriatrics and Gerontology Freiburg, University Medical Center Freiburg, Germany; d Department of Psychiatry and Psychotherapy, University Medical Center, Freiburg, Germany; e Dept. of Neuroradiology, Medical Center – University of Freiburg, Faculty of Medicine, University of Freiburg, Germany; f Neuroradiology, University Hospital Carl Gustav Carus, Dresden, Germany; g Neurology, University Hospital Carl Gustav Carus, Dresden, Germany; h Department of Neurology, Sankt Katharinen Hospital, Teaching Hospital of the Goethe University, Frankfurt, Germany; i Institute of Neuroradiology,Goethe University Frankfurt, Germany; j Department of Nuclear Medicine, University Medical Center Freiburg, Faculty of Medicine, University of Freiburg, Germany; k Department of Computer Science, University of Freiburg, Germany

## Abstract

The identification of pathological atrophy in MRI scans requires specialized training, which is scarce outside dedicated centers. We sought to investigate the clinical usefulness of computer-generated representations of local grey matter (GM) loss or increased volume of cerebral fluids (CSF) as normalized deviations (z-scores) from healthy aging to either aid human visual readings or directly detect pathological atrophy.

Two experienced neuroradiologists rated atrophy in 30 patients with Alzheimer's disease (AD), 30 patients with frontotemporal dementia (FTD), 30 with dementia due to Lewy-body disease (LBD) and 30 healthy controls (HC) on a three-point scale in 10 anatomical regions as reference gold standard. Seven raters, varying in their experience with MRI diagnostics rated all cases on the same scale once with and once without computer-generated volume deviation maps that were overlaid on anatomical slices. In addition, we investigated the predictive value of the computer generated deviation maps on their own for the detection of atrophy as identified by the gold standard raters.

Inter and intra-rater agreements of the two gold standard raters were substantial (Cohen's kappa *κ* > 0.62). The intra-rater agreement of the other raters ranged from fair (*κ* = 0.37) to substantial (*κ* = 0.72) and improved on average by 0.13 (0.57 < *κ* < 0.87) when volume deviation maps were displayed. The seven other raters showed good agreement with the gold standard in regions including the hippocampus but agreement was substantially lower in e.g. the parietal cortex and did not improve with the display of atrophy scores. Rating speed increased over the course of the study and irrespective of the presentation of voxel-wise deviations.

Automatically detected large deviations of local volume were consistently associated with gold standard atrophy reading as shown by an area under the receiver operator characteristic of up to 0.95 for the hippocampus region. When applying these test characteristics to prevalences typically found in a memory clinic, we observed a positive or negative predictive value close to or above 0.9 in the hippocampus for almost all of the expected cases. The volume deviation maps derived from CSF volume increase were generally better in detecting atrophy.

Our study demonstrates an agreement of visual ratings among non-experts not further increased by displaying, region-specific deviations of volume. The high predictive value of computer generated local deviations independent from human interaction and the consistent advantages of CSF-over GM-based estimations should be considered in the development of diagnostic tools and indicate clinical utility well beyond aiding visual assessments.

## Highlights

• The visual identification of atrophy is most accurate in the temporal lobe.
• Voxel-wise deviations of tissue volume from normal aging is easy to implement.
• Displaying voxel-wise deviations subjectively but not objectively aids readers.
• Voxel-wise deviations themselves show high agreement with expert readings.
• Information on tissue deviations should be provided with cerebral MRI scans.

## 1 Introduction

The accuracy of MRI-based diagnostics of neurodegenerative disorders depends on the level of expertise of the involved radiologists (Klöppel et al., 2008a). A key element in integrating imaging for the diagnosis is to recognize combinations of regions that show signs of pathological neurodegeneration. A recent study has shown that expert neuroradiologists can accurately identify a range of neurodegenerative disorders based on MRI, particularly when the information from multiple rating scales is integrated (Harper et al., 2016). However, outside specialized centers, where the majority of cases is seen, expert neuroradiologists are unavailable and therefore computer-aided analysis techniques may support the process of diagnosis.

Over the past decade, research in the field of computer-assisted diagnosis had a strong focus on multivariate pattern recognition methods that have successfully identified a wide range of pathological conditions (Klöppel et al., 2012). Although the integration into the routine of a memory clinic remains challenging (Klöppel et al., 2015), their ability to separate different types of neurodegenerative diseases from one another as well as from healthy aging and to predict the conversion to dementia in individuals with mild cognitive impairment has first been shown a decade ago (Adaszewski et al., 2013; Cuingnet et al., 2011; Davatzikos et al., 2011; Davatzikos et al., 2008; Dukart et al., 2013; Fan et al., 2008; Heister et al., 2011; Klöppel et al., 2008b; Misra et al., 2009; Teipel et al., 2007; Vemuri et al., 2009; Vemuri et al., 2008a; Vemuri et al., 2008b).

Despite the promising performance on study samples, several limitations of these methods exist. These supervised machine-learning techniques require training data from which they learn the diagnostic separation and can introduce non-obvious and non-trivial biases whenever training samples are not representative: Despite efforts for data sharing, it will remain difficult to fulfill that requirement e.g. for the various subtypes of e.g. Fronto-temporal dementia (FTD) and Alzheimer's Disease (AD) and when considering the presence of multiple pathologies in the same individual (Lim et al., 1999; Toledo et al., 2012) as well as MRI sequence parameters. High numbers of training samples and good control of technical and demographic factors (Abdulkadir et al., 2013; Abdulkadir et al., 2011) can reduce this problem to some extent, but the diagnostic accuracy in a clinical setting is significantly lower than in research samples (Klöppel et al., 2015).

Rather than relying on the diagnostic output of a computerized tool, this study aimed to evaluate the usefulness of computing voxel-wise the tissue specific deviation from the distribution in normal controls after controlling for e.g. age in order to provide the clinical rater with additional quantitative information. This approach is an application of well- established voxel-based-morphometry (VBM) (Ashburner and Friston, 2000) and a fast and robust strategy to obtain local grey matter (GM) and cerebro-spinal fluid (CSF) volumes of individual subjects. We performed two analyses to study usefulness. In the first, we assessed the added value of displaying deviations from the expected local tissue volumes along with the native image to aid raters in the visual assessment of MRI scans. We here defined usefulness as an increase in performance which was measured either as agreement with the reference atrophy rating, time required to perform the reading, and consistency of each individual reader when (unknowingly) seeing the same subject twice. The second analysis assessed the agreement of region-wise aggregated volume deviation scores directly with the gold standard rating. To this end, we computed receiver-operator-characteristics (ROCs) and report the positive and negative predictive value for prevalences, typically observed in a memory clinic.

Atrophy was rated in five anatomically defined brain regions per hemisphere, typically affected early in the course of neurodegenerative dementias. As in our previous work (Klöppel et al., 2008a) raters differed in their level of experience with the rating of cerebral MRI scans. All received a brief written instruction with examples how atrophy should be identified and how the voxel-wise volume deviation maps indicate atrophy. We expected experienced raters to show a higher agreement with the gold standard rating and that the colored maps would be the more useful the less experienced a rater is and improve agreement with the gold standard as well as intra-rater reliability. Although the display of additional information may require extra time to interpret, we expected this to be outweighed by less ambiguous information. While changes in GM volume are more directly related to disease-specific neurodegeneration, rating scales (e.g. medial temporal lobe atrophy score (Scheltens et al., 1992)) often explicitly include a widening of CSF-spaces as criterion. Additionally, and from a technical viewpoint, the borders between CSF are better defined compared to those between GM and white matter. We therefore included visualizations of deviations from local CSF volume in our analysis. We also expected that the visual presentation of volume deviations maps would increase the consistency of ratings of individual readers and the subjective level of diagnostic confidence. In line with previous work (Harper et al., 2016), we expected the highest agreement with the gold standard raters in the medial temporal cortex. We included subjects with dementia due to AD, FTD and dementia due to Lewy body disease (LBD) as well as healthy older individuals (HC). The group with LBD was included for consistency with our earlier work (Klöppel et al., 2015) and that of others (Harper et al., 2016) and although LBD-related changes on T1-weighted MRI are subtle.

## 2 Methods

### 2.1 Study data

Structural MRI from 120 subjects^3^ (30 HC, 30 AD, 30 FTD and 30 LBD) were acquired in Freiburg, Munich, and Leipzig or were taken from the Alzheimer's Disease Neuroimaging Initiative (ADNI) database (adni.loni.usc.edu) (Mueller et al., 2005). The ADNI was launched in 2003 as a public-private partnership, led by Principal Investigator Michael W. Weiner, MD. The primary goal of ADNI has been to test whether serial magnetic resonance imaging (MRI), positron emission tomography (PET), other biological markers, and clinical and neuropsychological assessment can be combined to measure the progression of mild cognitive impairment (MCI) and early AD. For up-to-date information, see www.adni-info.org.

In order to avoid a confound between diagnostic category and scanning parameters, ten cases with AD, LBD, ten controls, and six cases with FTD were provided from Freiburg with identical scanning parameters and supplemented with external data to fill each category. The overview of sociodemographic information is listed on Table 1.

**Table 1:** Sociodemographic characterization of the study cohorts.

	Healthy controls		Alzheimer's disease		Lewy-body dementia		Frontotemporal dementia	
	Mean	SD	Mean	SD	Mean	SD	Mean	SD
n (m/f)	13/17		14/16		14/16		11/19	
Age (years)	72.4	4.4	71.6	7.4	72.2	7.7	65.7	7.8
Education (years)	16.7	2.9	14.8	2.4	10.9	2.4	11.1	3.7
MMSE	29.0^a^	1.5	23.2	3.2	22.1	3.2	22.8	3.4

Note: SD = standard deviation, MMSE = Mini Mental Status Examination

a For *n* = 6, no MMSE was available but cognitive functioning was evaluated with the Montreal Cognitive Assessment (MoCA, Nasreddine et al. (2005)). MoCA scores were converted into MMSE scores according to Roalf et al. (2013).

Participants from all diagnostic groups showed clinical and biomarker patterns consistent with their respective diagnosis. Specifically, HC from Freiburg were included if their Montreal Cognitive Assessment (MoCA) score was ≥26 (Nasreddine et al., 2005) and their Beck's Depression Inventory-II (Beck et al., 1996) was ≤13. For the ADNI, HC were included if cognitive functioning above education adjusted cut-offs in the Logical Memory II subscale from the Wechsler Memory Scale–Revised was documented, their Geriatric Depression Scale was ≤6, their MMSE score was between 24 and 30 (inclusive), and their CDR was 0 (with a sum-of-box score of 0). We used an additional independent sample of healthy controls to estimate the normative range of GM and CSF volumes (see Section 2.4 for details).

Patients with AD all met clinical criteria for probable AD dementia with biomarker evidence. They all had clinical T1w MRI and had undergone 18fluorodeoxyglucose (18F-FDG) or amyloid PET. The biomarker patters clearly indicated AD pathology according to established criteria (McKhann et al., 2011). Patients with AD from the ADNI sample additionally had a CDR of 0.5–1. Patients with Lewy body disease all presented with two or more clinical core features and met criteria for probable LBD (McKeith et al., 2017) and, additionally, showed reduced ^18^F-FDG uptake (Minoshima et al., 2001; Yong et al., 2007). A subset of 20 cases has been previously reported (Perneczky et al., 2007). We recruited a mixed sample of cases with FTD. Patients with FTD met criteria (Neary et al., 1998) for either probable behavioural-variant frontotemporal dementia (bvFTD) with frontal and/or anterior temporal hypometabolism on ^18^F-FDG PET and frontal and/or anterior temporal atrophy on MRI (Rascovsky et al., 2011) or for semantic dementia (SD) that is characterized by predominant anterior temporal hypoperfusion or hypometabolism on PET (Gorno-Tempini et al., 2011). A subset of cases classified with FTD has been previously reported (Dukart et al., 2011; Frings et al., 2010).

### 2.2 Image acquisition

Sagittal T1-weighted 3D magnetization prepared rapid gradient echo (MPRAGE) sequences (approximately 1 × 1 × 1 mm^3^ resolution) were acquired in Freiburg, Leipzig and Munich on different types of 1.5 and 3 Tesla Siemens scanners, each with a standard head coil, or were obtained from the ADNI, being acquired at 1.5 or 3 Tesla magnetic field strength. The MPRAGE sequences were converted to NIfTI-2 format, and the filenames were pseudonymised before further processing.

### 2.3 Data processing

All T1 weighted images were processed identically. For the voxel-wise analysis, we generated spatially normalized probability maps of GM and CSF using SPM12 segmentation (Ashburner and Friston, 2005) and non-linear registration algorithms (Ashburner and Friston, 2011). Normalized and modulated images were smoothed with a 3 × 3 × 3 mm FWHM Gaussian kernel. The default setting was used for processing and the modulated warped tissues of GM and CSF after smoothing were used for the subsequent analyses.

### 2.4 Estimation of normalized local GM/CSF volumes and display of GM/CSF atrophy indices

At every voxel of each individual, we computed two volume deviation scores, one for GM and one for CSF. The score indicated the normalized deviation from the expected mean given the subject's age, sex, and intracranial volume. It was defined as the z-score of the residual GM/CSF after correction for covariates as follows. We adjusted the quantity of GM and CSF linearly for effects of age, sex, and total intracranial volume based on the reference population using ordinary least squares estimation of regression parameters and the residual variance as estimate of uncertainty. To this end, three hundred sixty-two healthy controls from the ADNI study were included to estimate healthy aging based on a large population and the expected GM/CSF volumes were subtracted from the measured volume (Dukart et al., 2011). This reference data was acquired on a variety of scanners from different vendors, field strengths, and with different receiver head coil configurations. The residual variance was computed using the same data and we assumed the variance to be homoscedastic, meaning that it was assumed to be equal regardless of the subject's characteristics such as age or sex. Formally, the atrophy scores *a*_*GM*_^(*v*,*s*)^ and *a*_*CSF*_^(*v*,*s*)^ for GM and CSF in the reference MNI space at voxel *v* of subject *s* were defined as

$$a_{GM}^{\left(v,s\right)}=-\frac{y_{\mathrm{GM}}^{\left(v,s\right)}-x^{s}{\hat{\beta }}_{\mathrm{GM}}^{v}}{{\hat{\sigma }}_{\mathrm{GM}}^{v}}\;\text{and}\;a_{\mathrm{CSF}}^{\left(v,s\right)}=+\frac{y_{\mathrm{CSF}}^{\left(v,s\right)}-x^{s}{\hat{\beta }}_{\mathrm{CSF}}^{v}}{{\hat{\sigma }}_{\mathrm{CSF}}^{v}}$$

where *s* and *v* indicate the subject and voxel location, respectively, y is the measured local volume of GM or CSF, *x* the predictors (age, sex, TIV), $\hat{\beta }$ the estimated parameters of the linear model, and $\hat{\sigma }$ the estimated residual variance. The parameters $\hat{\beta }$ were estimated with ordinary least squares method. Note that the parameter estimates at each voxel *v* were the same for all subjects since these scores and the parameters were estimated with coregistered GM/CSF tissue probability maps and were then transformed to native space for the visualization.

**Fig. 1:**
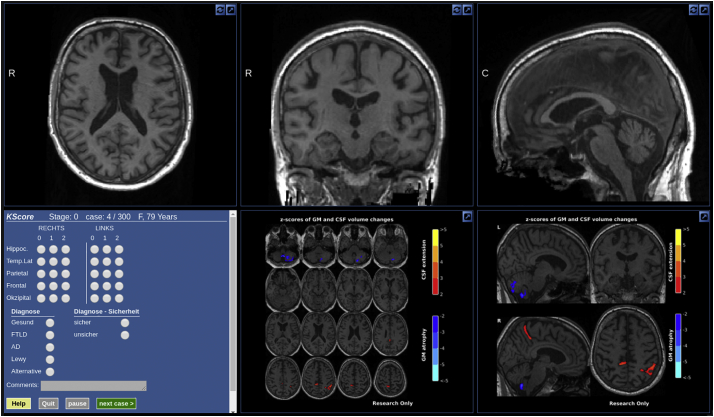
Web interface for readers showing T1 image, atrophy scores, rating questionnaire, and basic demographic information. The interface allowed to scroll through the slices of the structural MR image in all orthogonal views, to adjust the contrast and to increase each subpanel to full-screen. The sections showing the atrophy scores (bottom right on this panel) were static but could also be enlarged to fill the screen.

We assumed that low normalized local GM volumes and high normalized local CSF volumes were particularly informative to rate atrophy and discarded changes in the opposite direction. We chose a threshold of two standard deviations from which on we displayed deviations per voxel. Voxels with GM atrophy score larger than 2 (corresponding to z-scores smaller than −2) were displayed in four cold colours ranging from dark to light blue. Voxels with CSF-based deviation scores larger than 2 (corresponding to z-scores larger than 2) were displayed in four warm colours ranging from dark red to yellow. In the rare cases where voxels had high GM and high CSF atrophy scores simultaneously (partial volume effects), only the colour for GM atrophy was displayed. To further reduce clutter, only clusters of at least five voxels with atrophy scores above 2 were shown and only in voxels where the average local GM/CSF volume of the healthy population was above 0.5. An example of a resulting overlay is shown in Fig. 1.

### 2.5 Reading

The seven individual readers were presented with scrollable sections of the T1 image (Fig. 1) twice, either with or without visualization of atrophy scores of GM and CSF. We made the assumption that in general the readers would not recognize an already seen subject and remember the rating that they gave for the individual regions, thus that two readings of the same subject once with and once without visualization of atrophy scores would be independent. Furthermore, we made the assumption that seeing the same image twice with or twice without visualization of atrophy scores would be independent given a certain interval between both. Thus, to avoid an immediate repetition of the same image, images were presented in two blocks without repetitions in the same block. To measure intra-rater reliability, 30 scans with and 30 scans without atrophy scores were shown twice in the same block. In total, each individual reader reviewed 300 scans and 3000 single region ratings (5 regions, 2 hemispheres).

No clinical information apart from age and sex was provided to the readers. Atrophy in ten regions had to be rated on a scale with three levels (0 = normal; 1 = borderline atrophy; 2 = pathological atrophy). The instructions were limited to four pages that were submitted to the raters without further comments. Each page showed one example for each level of atrophy of one or two regions. Examples of temporo-lateral and frontal regions were shown on the same page and examples of the remaining three regions were shown on separate pages. The scores were adapted from established rating scales. Hippocampus atrophy was adapted from the medial temporal atrophy (MTA) score proposed by Scheltens et al. (1992). MTA scores ≤ 1 were translated to no atrophy, MTA score = 2 was translated to borderline atrophy, and MTA score ≥ 3 was translated to pathological atrophy. The parietal atrophy rating followed the rating proposed by Koedam et al. (2011). Koedam score 0 and Koedam score 1 were translated to no atrophy and borderline atrophy, respectively. Koedam score two or larger were translated to pathological atrophy in our study. Frontal and lateral temporal atrophy was rated following Davies et al. (2009). Scores 0 and 1 were equivalent in the original and our study and higher scores in the original scale were translated to pathological atrophy in our study. As there was no established rating scale for occipital atrophy available, we adapted the principle of evaluating prominent structures of the subarachnoid-space and ventricular system as used in the previously mentioned rating scales. As the calcarine fissure is the major CSF-containing structure within the occipital lobe MRI examples of a normal appearing, a slightly widened, and a definitely widened calcarine fissure were added to the instructions. As there was no established rating scale for occipital atrophy available, we adapted the principle of evaluating prominent structures of the subarachnoid-space and ventricular system as used in the previously mentioned rating scales. As the calcarine fissure is the major CSF-containing structure within the occipital lobe MRI examples of a normal appearing, a slightly widened, and a definitely widened calcarine fissure were added to the instructions.In addition to the atrophy rating, one of four mutually exclusive diagnoses had to be picked. Responses on the diagnostic decision will be reported separately. All fields had to be completed before the next subject was shown and raters could not go back to previously presented scans. Samples with and without deviation maps were shown in a random order fix across readers. As gold standard raters did not see z-scores, they received all images in one block of 150.

Both gold standard raters were trained neuro-radiologists with >15 years of experience in the field. The other raters deliberately had a wide range of expertise ranging from third year of specialization to 10+ years of experience as fully trained doctors.

To test whether less experienced readers would benefit more from displayed deviation maps, we asked them to indicate the number of brain scans for which they would rate the level of atrophy in a normal workweek. All readers received a brief manual which explained the orientation of the images (left/right) and that cold colours would indicate GM loss and warm colours an increase in local CSF volume. Next, the manual explained the use of the response field seen on the left side of Fig. 1. Readers were also reminded that the age of each subject was displayed. We presented a set of seven additional scans (not included in the evaluation set) to familiarize each reader with the system. After completing all ratings, readers were asked to indicate if they considered the display of voxel-wise deviations helpful in respect to speed and accuracy when reaching a decision on atrophy and diagnosis.

### 2.6 Evaluation of the added value of displaying computer assisted atrophy reading

We studied potential benefits of displaying atrophy scores alongside the plain structural MRI in the three categories accuracy, speed, and retest-reliability.

To quantify the agreement between each individual rater and the gold standard reading, we first excluded samples which one gold standard reader rated as normal (0) and the other rated as clearly pathological (2). Of note, the remaining regions of the scan remained in the analysis and no regions were excluded for analyses of speed and intra-rater agreement detailed below. Next, we defined three categories: full agreement required both gold standard-readers and the individual reader to exactly agree. Partial agreement additionally included cases when the individual rater agreed with one of the gold standard readers. We considered the agreement to be at chance level at 20% for full agreement (3 out of 15 valid combinations of ratings) and at 47% (7 out 15) for the more lenient criterion (i.e. partial or full agreement). Cases for which the individual rater disagreed with both gold standard readers where defined as no agreement.

We used a paired *t*-test to identify a difference in accuracy with and without atrophy scores. In cases that were presented twice as part of the intra-rater reliability analysis, we used only the ratings of the first presentation for the evaluations of accuracy. Although the atrophy reading and the computation of the agreement of the individual readings with the gold standard was done separately for left and right hemisphere, we pooled the results over both hemispheres to simplify the presentation of the results.

We performed a Pearson correlation to test for the expected positive association between the level of previous experience in MRI reading and the agreement with the gold standard and used Fisher's r2z test to identify significant differences in the regression slope. Specifically, with ratings based on native MRI, we expected more experienced raters to perform better but that this correlation would be absent or weaker when atrophy scores are displayed alongside the native MRI.

The time to complete the reading of a single volume was registered during the experiment and included the MRI based differential diagnosis. Readers had the possibility to pause the reading which resulted in a black screen and interrupted the timing. The participants were not given any instructions about how quickly to finish.

Retest-reliability was assessed by showing a random subset of 30 volumes with and 30 volumes without visualization of deviation maps in both blocks. Based on the ratings of the repeated volumes, we assessed the test-retest agreement using Cohen's Kappa (*κ*) with quadratic weight (Cohen, 1968) to account for the ordinal scale of the atrophy codes. We computed *κ* for the repeated ratings once with and once without visualization of the z-score deviation maps. Cohen's *κ* is usually used to measure inter-rater reliability due to the assumption of independent observations. We minimized the dependency of two observations performed by the same reader rating the same sample by using the two-block design detailed in Section 2.5. However, we cannot exclude the possibility of dependencies, thus we acknowledge that the estimation of the intra-rater agreement by the measure *κ* is possibly too high. We also report the intra-rater reliability as percent exactly identical ratings as a more intuitively accessible measure that, however, is insensitive to the extend of the disagreement and the frequencies of ratings.

### 2.7 Usefulness of atrophy scores alone

**Fig. 2:**
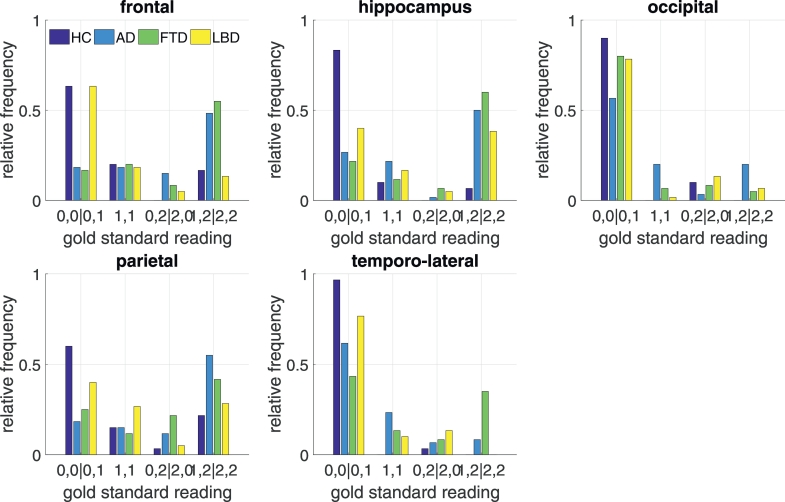
Distribution of atrophy readings per region and diagnostic category by the two gold standard readers. Regions which one reader identified as normal and the other as borderline (0,1) entered the same category as two normal ratings (0,0). Accordingly pathological atrophy identified by just one reader (1,2) was assigned the same category as two atrophy ratings (2,2).

Besides evaluating the benefits of the z-scores in assisting the human visual reading, we were interested how much value these scores would provide by themselves in informing about region-specific atrophy. For this analysis we focused on cases with a clear rating result and excluded regions where the gold standard readers fully disagreed and those where both agreed that the atrophy was borderline. Regions with one gold standard rating “zero” and another rating of “one” were graded as “zero”, one rating of “one” and another rating of two lead to an overall rating of two. Then, we computed for both tissue types (GM and CSF) the volume of voxels with deviations of more than two standard deviations separately for each region in each hemisphere to express the grade of atrophy in that particular region. Two standard deviations were used as cut-point when visually indicating deviations to the individual raters in the first part of the study and thus were kept for consistency. We report receiver operator characteristics (ROC) curves as well as the positive predictive value (PPV), negative predictive value (NPV), sensitivity (SE) and specificity (SP) at the threshold corresponding to a maximum of the product of SE and SP. To rate the clinical usefulness, we also report the rate of positive or negative predictions (RPP, RNP). The RPP/RNP are the proportions of the population that have a score higher/lower than the threshold for which the PPV/NPV is reported. The RPP/RNP thus indicate the proportion of cases for which the presence/absence of atrophy can be predicted with the reported PPV/NPV. Given the high prevalence of relatively rare dementia cases in our sample, the PPV would be unrealistically high. We thus computed PPV and NPV for prevalences expected in a memory clinic (Alladi et al., 2011; Claus et al., 2016). Specifically, we assumed the following distribution of HC: 34%, AD: 38%, FTD: 19%, LBD: 9%. For each region and diagnosis, we derived the prevalence of atrophy from the ratings of our gold standard readers as reported in Fig. 2. The prevalence of diagnoses and the diagnosis-dependent proportion of atrophy in each region were combined to yield for each region the expected proportion of atrophy as

$$p\left(\text{atrophy}|\text{region}\right)=p\left(\mathrm{AD}\right)p\left(\text{atrophy}|\mathrm{AD}\right)+p\left(\mathrm{FTD}\right)p\left(\text{atrophy}|\mathrm{FTD}\right)+p\left(\mathrm{LBD}\right)p\left(\text{atrophy}|\mathrm{LBD}\right)+p\left(\mathrm{HC}\right)p\left(\text{atrophy}|\mathrm{HC}\right)$$

where *p*(*D*) indicates the expected proportion of cases with diagnosis *D* as given by the field of application and *p*(atrophy|*D*) indicates the proportion of atrophy given the diagnosis *D* as found in this study. The positive predictive value of atrophy for the hypothesized clinical (rather than study-specific) population thus was

$$\mathrm{PPV}\left(\text{region}\right)=\frac{\mathrm{SE}\left(\text{region}\right)∙p\left(\text{atrophy}|\text{region}\right)}{\mathrm{SE}\left(\text{region}\right)∙p\left(\text{atrophy}|\text{region}\right)+\left(1-\mathrm{SP}\left(\text{region}\right)\right)∙\left(1-p\left(\text{atrophy}|\text{region}\right)\right)}$$

where *SE* (sensitivity) *SP* (specificity) were chosen at the atrophy score threshold that maximized the product of sensitivity and specificity.

## 3 Results

All raters completed the full data set. Subjectively, individual raters found the display of voxel-wise deviations helpful to increase the ease and speed of their rating.

Fully opposing atrophy ratings (one gold standard reader rated normal, “zero”, the other pathological atrophy, “two”) were submitted in 7.5% out of all 1200 individual regions.

Fig. 2 displays the distribution of ratings by the two gold standard raters. As expected, atrophy was far more frequently detected in samples from patients with AD and FTD, conditions with a clear pattern of atrophy detectable on MRI. Conversely, the frequency of atrophy in LBD and the consensus of its distribution was far lower. Detection of pathological atrophy was rarest in HC. Between the two gold standard raters and across all ratings, a full consensus was reached in over one third of all regions, reaching over 70% when the more liberal criterion (partial or full agreement) was applied. Agreements between the gold standard raters were highest for the hippocampus and lateral temporal cortex and low for frontal and occipital cortex.

**Fig. 3:**
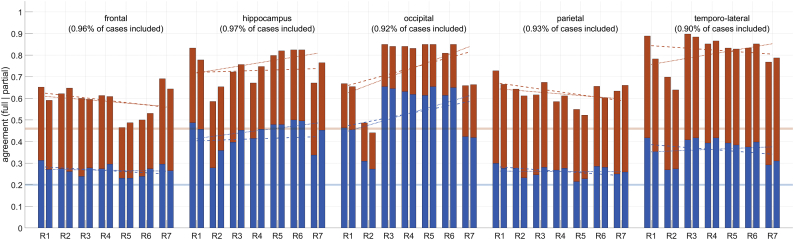
Accuracy of atrophy detections in relation to the gold standard reading. Blue indicates the accuracy using a strict definition of agreement while red displays accuracy levels with a more liberal criterion (see main text for detail). The reported regions are indicated above each panel. Numbers in brackets indicate the percentage of cases remaining after excluding those with conflicting ratings of the two gold standard readers. The readers (R1-R7) are ordered by increasing experience from left to right. For each reader, the left bar indicated performance without and the right bar with z-scores. The trendlines indicate qualitatively the relation between experience and accuracy without (dashed trendlines) and with (solid trendline) atrophy scores. The transparent horizontal blue and red line denote the chance level 0.2 for full and 0.46 partial accuracy, respectively.

When comparing individual ratings to the gold standard (Fig. 3) we found a similar pattern. Agreement was high for hippocampus, occipital lobe and temporo-lateral regions but lower for frontal and parietal cortices. Encouragingly, and irrespective of the criterion chosen for agreement, inter-rater agreement was consistently above chance level.

**Fig. 4:**
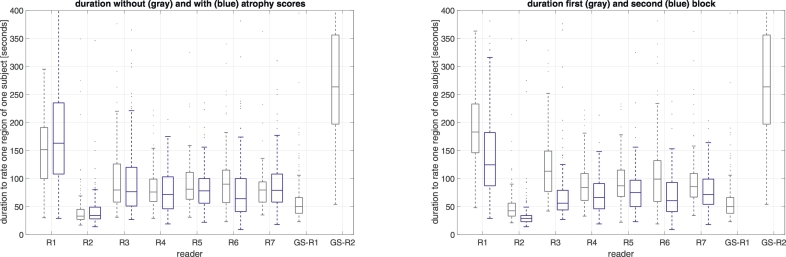
Left panel: Boxplots of individual reading times with (blue) and without (grey) atrophy score overlay. R1..R7 indicate the seven readers and GS-R1 and GS-R2 are the two gold standard readers. Note that GS-R1 and GS-R2 did not see the atrophy score maps. Right panel: Boxplots of individual reading times of first (grey) and second (blue) presentation. Note that GS-R1 and GS-R2 performed all readings in a single block.

All readers were significantly faster when rating images of the second block, probably due to training effects. On average, the time necessary to rate one volume was reduced from 110.8 s to 76.4 s (−31%). Average time to complete one reading did neither increase nor decrease significantly with the additional display of atrophy scores (Fig. 4).

The intra-rater agreement measured by Cohen's Kappa (κ) of the individual readers was often higher than that of the two gold standard raters. The display of atrophy scores improved the intra-rater agreement by 0.13 (range : 0.57 < κ < 0.87) and five out of seven readers benefitted from the z-scores (Table 3).

The level of previous experience did not correlate with the diagnostic accuracy, neither with nor without the display of atrophy scores (qualitatively shown on Fig. 3, Pearson correlation *p* > .05 (data not shown)).

**Fig. 5:**
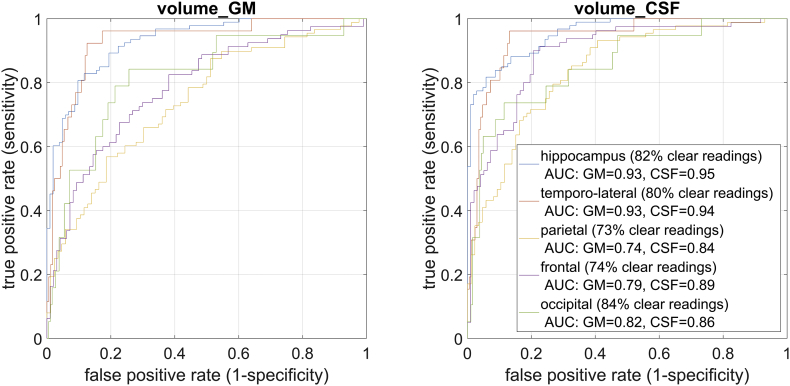
Receiver-operator curves for each of 5 anatomical regions when pooled across both hemispheres separately for z-Scores indicating either grey or white matter volume with atrophy scores larger than 2. We focused on cases with clear readings which were rated by both gold standard readers unanimously as normal or atrophic. The percentage of cases fulfilling that criterion is reported in brackets (clear readings).

When evaluating the ability of z-scores to directly detect atrophy independently from the individual rater, we found largest AUC for both GM and CSF for temporo-lateral and hippocampus atrophy. Across all regions, CSF based atrophy scores lead to a generally better discrimination (Fig. 5). To evaluate the performance of these classifiers in a hypothetical clinical setting, we used the known frequency of each diagnostic group in a memory clinic and the frequency of region specific atrophy per diagnostic group as identified by two gold standard raters (Fig. 2). The resulting prevalences are listed on Table 2. The evaluation of the ability of the automated method to correctly identify atrophy is depicted in Fig. 5. The high values for both SE and SP in the hippocampus and temporo-lateral lobe area underscore the more reliable rating of this regions.

**Table 2:** Characteristic of classification performance based on the region-wise volumes with atrophy scores above 2 for grey matter (GM) and cerebro-spinal fluid (CSF) evaluated using the non-ambiguous readings only. The fraction of expected unambiguous readings is denoted %unambiguous. The prevalence indicates the expected fraction of atrophy in each region for the population expected in a hypothetical memory clinic setting. Positive and negative predictive values (PPV, NPV) as well as the rate of positive predictions (RPP) are reported for the assumed prevalences. SE: sensitivity; SP: specificity.

	GM		CSF		Expected population		GM			CSF		
	SE	SP	SE	SP	Prevalence	%Unambiguous	PPV	NPV	RPP	PPV	NPV	RPP
Hippocampus	0.83	0.88	0.82	0.94	0.46	0.82	0.86	0.86	0.41	0.92	0.86	0.38
Temporo-lateral	0.92	0.87	0.96	0.87	0.13	0.8	0.52	0.99	0.24	0.52	0.99	0.25
Parietal	0.57	0.81	0.8	0.73	0.53	0.73	0.78	0.62	0.34	0.77	0.76	0.58
Frontal	0.68	0.77	0.9	0.79	0.51	0.74	0.75	0.7	0.47	0.82	0.88	0.6
Occipital	0.84	0.74	0.74	0.89	0.12	0.84	0.3	0.97	0.29	0.46	0.96	0.17

**Table 3:** Intra-rater consistency of atrophy reading with and without atrophy score maps evaluated by Cohen's Kappa statistics (κ) and by percent agreement (%a, defined as exactly identical scores) between test and re-test.

Reader	Native MRI (***κ***/ % ***a***)	Native MRI + atrophy score map (***κ***/ % ***a***)
GS-R1	0.65/67	n.a.
GS-R2	0.68/73	n.a.
R1	0.72/70	0.87/82
R2	0.37/56	0.67/64
R3	0.61/73	0.53/0.71
R4	0.66/0.77	0.83/0.84
R5	0.56/0.79	0.55/0.81
R6	0.63/0.79	0.76/0.82
R7	0.45/64	0.71/69

The population independent performance measures (i.e. SE and SP) of the z-scores as well as the population dependent measures (i.e. PPV and NPV) based on the optimal threshold obtained from the ROCs (Fig. 5) are listed in Table 2. As expected, high NPV but low PPV are reported for regions rarely affected by atrophy in the hypothetical memory clinic sample (Table 2). As a consequence of the superior test characteristics, performance was generally better when based on CSF atrophy scores.

## 4 Discussion

Our study investigated a potential beneficial effect of computing voxel-wise quantitative deviations of GM and CSF volumes to aid visual readers in the detection of atrophy and to directly conclude on the presence or absence of atrophy.

The pattern of atrophy as detected by the gold standard raters largely mirrored the expected disease specific pattern. Of note, the hippocampus was frequently rated as borderline or atrophic in cases with FTD and even more so than in cases with AD. This same pattern has been reported for other rating studies (Harper et al., 2016) and is well in line with VBM studies revealing no differences between FTD and AD in e.g. the hippocampus region but only in the parietal cortex and temporo-parietal junction (Du et al., 2007). Fig. 2 indicates that the distribution of detected atrophy would often not allow a separation between FTD and AD on the one hand and between LBD and HC on the other. Overall, accuracy ratings of the seven individual readers were satisfactory and higher in areas such as the hippocampus compared to e.g. the frontal or parietal lobe, again well in line with previous work (Harper et al., 2016). The readers were instructed to rate pathological versus non-pathological atrophy and therefore needed to account for age-related structural changes.

We found no beneficial effect of displaying z-scores, not even for relatively unexperienced readers (trendlines in Fig. 3). This is in some way surprising and contradicts the beneficial effect of the z-scores on time and accuracy subjectively perceived by the raters themselves. The relatively poor agreement of the two gold standard readers (Fig. 2) in regions such as the parietal cortex may have decreased our sensitivity to detect a benefit of the z-scores. Furthermore, since the *κ* statistics takes the frequency of levels into account, naïvely rating all cases with the majority class would achieve a high accuracy but not a high *κ* value. An alternative reason for the failure of detecting beneficial effects of z-scores is the possibility that z-scores could encourage false positive or negative atrophy ratings. *Z*-score deviations were not scaled to mimic the gold standard raters and the individual raters did not know the optimal cut-point. In any case, the increase in intra-rater agreement with the display of z-scores and regional statistics clearly shows that those influenced the atrophy rating and were not ignored. However, an increase in intra-rater agreement on its own is not useful if not associated with more accurate ratings. While VBM is a relatively robust method, there are several sources that can introduce a bias. Image artefacts are generally relevant here but potentially less so in our data set given that it was largely derived from well controlled studies. However, imperfect image registration or variations in the individual cortical folding pattern could explain discrepancies between z-scores and an experienced rater and illustrate that z-scores may not always indicate atrophy in a biological sense. Of note, major errors in image registration are become obvious to the raters as deviation maps were overlayed on the individual brain scan. Although both hemispheres were rated separately, we pooled across both hemispheres. This is justified by studies showing no systematic asymmetry of atrophy across neurodegenerative diseases (Minkova et al., 2017). On the other hand, individual cases may show asymmetric atrophy e.g. the language subtype of FTD is typically associated with atrophy of the left temporo-lateral cortex and pooling across hemispheres may have levelled out the clearer left-hemisphere readings.

We found no effect of displaying deviation scores on the speed of rating. Displaying voxel-wise deviations increase the amount of information which could require extra time. On the other hand, provided information could increase clarity of the rating. Our results indicate that these two factors may outweigh each other. As mentioned already, readers subjectively perceived an increase in rating speed resulting from the displayed scores which may indicate that they would be more willing to rate scans in an actual clinical setting when z-scores were displayed alongside.

When evaluating the usefulness of region-specific average volume deviations irrespective of the individual readers, we found large AUCs particularly when deviations where computed from the CSF segment (Fig. 5). The CSF segment poses more clearly defined boundaries compared to the GM-segment which may explain favourable performance. While several visual rating scales already include an evaluation of CSF spaces (Davies et al., 2009; Koedam et al., 2011; Scheltens et al., 1992), our study indicates that changes in the CSF segment should also be the basis for the automated analysis of GM loss. When applying the classifier to a hypothetical clinical setting, the resulting PPV and NPV were frequently above 0.9 and may indicate clinical usefulness depending on the exact set of consequences. Although all regions performed well (minimal AUC for CSF-based classifier = 0.84), best performance was reached for the lateral and medial temporal lobes. Of note, this analysis was restricted to regions either clearly normal or clearly atrophic as judged by the gold standard raters which lead to the exclusion of up to 27% of cases for regions such as the parietal and frontal cortex. It is likely that the exclusion of borderline or inconsistently rated cases resulted in more optimistic estimates for the AUCs and the resulting PPV and NPV values an effect probably made more severe by the employed study sample. We used the volume of voxels with a z-score deviating by more than two standard deviations as a metric to quantify region specific deviations in a single variate. This metric was chosen as it resembles the visualization provided to the human raters where we also used a minimum deviation of two standard deviations.

In summary, we found beneficial effect from displaying voxel-wise deviations scores to aid human visual reading of cerebral MR-scans to be limited to intra-rater reliability and the subjective speed and accuracy. On the other hand our results on the detection accuracy of z-scores on their own motivate the automated analysis of z-scores based on the CSF segment separately from a visual rating. The exact threshold should be based on the local setting where a high sensitivity may outweigh an increasing number of false positives.

## Footnotes

3 One case with FTD appeared twice in the data set. The two scans from different visits had different patient identifiers. This was coincidently found after the analyses were completed.
